# Purging human ovarian cortex of contaminating leukaemic cells by targeting the mitotic catastrophe signalling pathway

**DOI:** 10.1007/s10815-021-02081-9

**Published:** 2021-03-16

**Authors:** Lotte Eijkenboom, Callista Mulder, Bert van der Reijden, Norah van Mello, Julia van Leersum, Thessa Koorenhof-Scheele, Didi Braat, Catharina Beerendonk, Ronald Peek

**Affiliations:** 1 Department of Obstetrics and Gynaecology, Radboud University Medical Centre, Nijmegen, The Netherlands; 2 Department of Reproductive Biology, Amsterdam University Medical Centre, University of Amsterdam, Amsterdam, The Netherlands; 3 Department of Laboratory Medicine, Laboratory of Haematology, Radboud Institute of Molecular Life Sciences, Nijmegen, The Netherlands; 4 Department of Obstetrics and Gynaecology, Amsterdam University Medical Centre, Vrije Universiteit, Amsterdam, The Netherlands

**Keywords:** Aurora kinases, Cryopreservation, GSK1070916, Myeloid leukaemia, Ovarian cortex, Purging

## Abstract

**Purpose:**

Is it possible to eliminate metastasised chronic myeloid leukaemia (CML) and acute myeloid leukaemia (AML) cells from ovarian cortex fragments by inhibition of Aurora B/C kinases (AURKB/C) without compromising ovarian tissue or follicles?

**Methods:**

Human ovarian cortex tissue with experimentally induced tumour foci of CML, AML and primary cells of AML patients were exposed to a 24h treatment with 1 μM GSK1070916, an AURKB/C inhibitor, to eliminate malignant cells by invoking mitotic catastrophe. After treatment, the inhibitor was removed, followed by an additional culture period of 6 days to allow any remaining tumour cells to form new foci. Ovarian tissue integrity after treatment was analysed by four different assays. Appropriate controls were included in all experiments.

**Results:**

Foci of metastasised CML and AML cells in ovarian cortex tissue were severely affected by a 24h ex vivo treatment with an AURKB/C inhibitor, leading to the formation of multi-nuclear syncytia and large-scale apoptosis. Ovarian tissue morphology and viability was not compromised by the treatment, as no significant difference was observed regarding the percentage of morphologically normal follicles, follicular viability, glucose uptake or in vitro growth of small follicles between ovarian cortex treated with 1 μM GSK1070916 and the control.

**Conclusion:**

Purging of CML/AML metastases in ovarian cortex is possible by targeting the Mitotic Catastrophe Signalling Pathway using GSK1070916 without affecting the ovarian tissue. This provides a therapeutic strategy to prevent reintroduction of leukaemia and enhances safety of autotransplantation in leukaemia patients currently considered at high risk for ovarian involvement.

**Supplementary Information:**

The online version contains supplementary material available at 10.1007/s10815-021-02081-9.

## Introduction

With the increase in overall 5-year survival rates in cancer [8, 70] due to advances in early detection and treatment, the effect on fertility of anti-cancer treatment has been gaining growing attention. As both radiotherapy and chemotherapy regimens can lead to subfertility or even infertility [31, 67, 73], the options for fertility preservation should be discussed before start of therapy.

Nowadays, multiple options are available for fertility preservation in women, however, for prepubertal girls and women who cannot delay start of their anti-cancer treatment ovarian tissue cryopreservation (OTC) is currently the sole option [13]. Ovarian cortex fragments are generally cryopreserved before start of anti-cancer therapy or during the first remission phase and used for restoration of fertility [69]. After autotransplantation, the ovarian follicles in the graft resume development and ovarian activity are restored in more than 93% of the cases allowing these women to conceive naturally or through assisted reproductive technology [18, 20, 21]. The first child was born in 2004 with the aid of OTC and the number of live births has been steadily rising and currently exceeds 130 [19, 29]. Live birth rates per autotransplantation are ranging from 23% up to 69% [20, 71].

Despite these excellent results, there are serious concerns regarding safety of autotransplantation of frozen-thawed ovarian tissue in former cancer patients. As the ovarian cortex tissue is generally obtained prior to start of the therapy, possible presence of metastasised malignant cells in the tissue may lead to reintroduction of the malignancy after autotransplantation. Many cancer types are known to metastasise to the ovary and especially hematologic malignancies are considered high risk [3, 16, 17, 66]. The presence of leukaemic cells in ovarian tissue from patients has been demonstrated by PCR analysis, multicolor flow cytometry and xenografting to immunodeficient mice [1, 9, 57, 72, 83].

Several strategies to circumvent the possibility of reintroducing malignant cells with the graft and increase the safety of OTC are currently pursued. These are based on separating the follicles from the possibly contaminated stromal cell compartment, and include in vitro maturation of primordial follicles [54, 78] and in vivo grafting of isolated pre-antral stage follicles in an artificial ovary, a decellularised ovarian matrix or by xenografting [2, 12, 48, 63].

In addition to these strategies, we have recently shown ex vivo purging of ovarian cortex tissue is a promising technique to prevent reintroduction of cancer by eradicating contaminating malignant cells, without affecting the ovarian tissue and follicles [59]. Foci of rhabdomyosarcoma cells were completely eliminated from ovarian cortex fragments by suppression of the Hippo signalling pathway using Verteporfin as an inhibitor of YAP/TAZ oncoproteins. However, tissue contaminated with myeloid leukaemia was not completely purged by treatment with Verteporfin.

Purging was also recently applied on ovarian cortex tissue from patients with acute lymphocytic leukaemia (ALL) using dexamethasone, known for its anti-leukaemic activity, but was found to be ineffective in purging ovarian cortex from contaminating malignant cells [9]. This, together with our previous findings, clearly indicates that different malignancies require different purging regimes [59].

Uncontrolled proliferation is a universal property of malignant cells, while ovarian cortex tissue, including the primordial follicles, is essentially mitotically silent [34]. Oncosuppression by targeting the mitotic catastrophe signalling pathway leads a cell to an irreversible antiproliferative fate of death or senescence and could therefore be a promising purging strategy [52, 80]. Although this pathway can be activated by several mechanisms, the inhibition of specific kinases such as the Aurora protein kinases has been shown to be very effective in disrupting mitotic progression. Aurora kinases are a family of serine/threonine kinases that are indispensable to cell division [7]. Dysregulation of Aurora kinases leads to mitotic abnormalities, failure of cytokinesis leading to formation of syncytia and ultimately to apoptosis [24, 27, 35]. Human malignancies frequently overexpress Aurora kinases, making them attractive and logical targets for purging [58].

In this paper, we have investigated the use of GSK1070916, a reversible and ATP competitive inhibitor of Aurora B and Aurora C kinases (AURKB/C), to purge ovarian cortex tissue fragments contaminated with foci of chronic myeloid leukaemia (CML) or acute myeloid leukaemia (AML) from cell lines and of primary cancer cells from patients with AML. We provide evidence that GSK1070916 is effective in inducing mitotic catastrophe in malignant cells of both CML and AML origin without compromising the integrity of the ovarian cells, including the follicles. Preventing reintroduction of malignancies before autotransplantation of ovarian cortex tissue by the pharmacological inhibition of Aurora kinases could therefore provide an effective therapeutic strategy for enhancing the safety of OTC.

## Materials and methods

### Selection of tumour cell lines

The cell lines used for this study consisted of CML: JURL-MK1, K562 and MEG-01, and AML: KG-1a, NB4 and NOMO-1 (Table 1). These cell lines represent various types of myeloid leukaemia and have been widely used for in vitro and in vivo studies [23, 43, 53, 82]. Cell lines were obtained from ATCC (Manassas, VA, USA) and DSMZ (Braunschweig, Germany). All cell lines were cultured in RPMI (BioWhittaker, Lonza, Basel, Switzerland) supplemented with 10% Fetal Bovine Serum (FCS, Gibco, Life Technologies, Carlsbad, CA, USA) and 40 μg/mL Gentamycin (Centrafarm, Etten-Leur, The Netherlands) and kept in log phase.

**Table 1:** Characteristics of cell lines in suspension culture. Morphological characteristic of human CML [JURL-MK1, K562 and MEG-01] and AML [KG1A, NB4 and NOMO-1] cell lines grown in suspension culture

Cell line	Disease	Differentiation stage	Morphology in suspension culture	Supplier	Doubling time in suspension (h)
JURL-MK1	CML	blast crisis	single round cells and small clusters	DSMZ	48
K562	CML	blast crisis	single round cells	ATCC	30-40
MEG-01	CML	megakaryoblast	single round cells or in small clusters, some cells slightly adherent	ATCC	35
KG-1a	AML	undifferentiated	single round cells, few giant cells, some cells slightly adherent	ATCC	50
NB4	AML	promyelocytic	polymorphic single cells, very few giant cells	DSMZ	35-45
NOMO-1	AML	myeloid	single round cells	DSMZ	35

### Primary cells

Cryopreserved primary AML cells were obtained from four AML patients (aged 64–80). Cells were thawed in FCS containing DNAseI (67 μg/mL, Sigma-Aldrich, Saint Louis, MO, USA), Heparin (17 U/mL, pharmacy Radboudumc, Nijmegen, The Netherlands) and MgSO_4_ (7 nM, Boom, Meppel, The Netherlands), incubated for 10 min at room temperature and washed twice with Phosphate Buffered Saline (Sigma-Aldrich, Saint Louis, MO, USA) containing 1% FCS. Cells were resuspended in Dulbecco’s Modified Eagle’s Medium (DMEM, BioWhittaker, Lonza, Basel, Switzerland) with 10% FSC.

### Collection of human tissue

Intact ovaries were obtained after informed consent from transgender men undergoing oophorectomy as part of their gender-affirming surgery (aged 18–24). Immediately after surgery, the ovaries were submerged in cold L15 medium (Lonza, Basel, Switzerland) and transported on ice to the laboratory. Cortical fragments of 10 × 10 × 1 mm were prepared and cryopreserved within 4 h after surgery according to clinical standards using previously described methods [61]. Testicular tissue was obtained from a patient undergoing a testicular sperm extraction and after the collection of spermatozoa was completed.

### Western blot

Expression of AURKB and AURKC in myeloid leukaemic cells grown in suspension and enzymatically digested human ovarian cortex tissue was examined by SDS-PAGE and Western blot analyses. Human testicular tissue, known for high level expression of AURKC [10, 42], was used as a control. In brief, cells were harvested, counted and resuspended in SDS-PAGE sample buffer containing 100 mM Dithiothreitol. Approximately 50 μg of total cell protein was subjected to standard 10% SDS-PAGE. After electrophoresis, samples were transferred to nitrocellulose membranes (GE Healthcare, Chicago, IL, USA) and probed with primary antibodies directed against AURKB (1:2000, Invitrogen, Carlsbad, CA, USA) or AURKC (1:500, Bioss Antibodies, Woburn, MA, USA), followed by incubation with a labelled secondary antibody (Cell Signalling Technology, Danvers, MA, USA). Proteins were visualised by chemiluminescence using Odyssey CLx Imaging system (LI-COR Biosciences, Lincoln, NE, USA). As a control, an antibody against Glyceradehyde 3-phosphate dehydrogenase (GAPDH, Abcam, Cambridge, UK) was used.

### Tumour induction

After thawing according to clinical standards [61], ovarian cortex fragments were micro-injected with exponentially growing CML cells (JURL-MK1, K562, MEG-01), AML cells (KG-1A, NB4, NOMO-1) or primary AML cells to induce micro metastases. The procedure was performed as previously described [61] using forceps and a 30-gauge needle with 0.3 mm outer diameter (Henke-Sass, Wolf GmbH, Nörten-Hardenberg, Germany). After tumour cell injection, the ovarium cortex fragments were cultured for 3 days to allow formation of small tumour foci.

### Ex vivo purging

To select the optimal concentration for GSK1070916 (GlaxoSmithKline, Brentford, UK) in relation to tumoricidal activity, cancer cell lines were cultured in suspension in a 24-well plate for 24 h with increasing concentration (100 nM, 1 μM and 10 μM) of GSK1070916 or an equivalent concentration of solvent (dimethylsulfoxide, DMSO; WAK-Chemie Medical GmbH, Steinbach, Germany) and examined by light microscopy. In all subsequent purging experiments, ovarian cortex tissue was exposed to 1 μM GSK1070916916 or an equivalent concentration of solvent. After tumour formation, the cortex fragments were cut in half. One half was used for purging by 24 h incubation in 5 mL of culture medium consisting of DMEM supplemented with 10% FCS and 40 μg/mL Gentamycin containing 1 μM GSK1070916 dissolved in DMSO. The other half was used as a control and exposed to an equivalent concentration of solvent. After 24 h of treatment, the fragments were transferred to a 6-well plate and were subjected to four subsequent 10 min washes in 5 mL of fresh DMEM to remove the inhibitor and solvent. During washing, the fragments were placed on a shaking table at 37 °C for optimal diffusion. Next, fragments were cultured for an additional 6 days in 5 mL of culture medium to allow any remaining tumour cells to form new foci. The purging protocol was performed with ovarian cortex tissue of three subjects for all CML and AML cell lines. To confirm the effectiveness of the purging protocol on primary cells, the experiment was repeated with cells from four AML patients.

### Histological examination

Fragments were fixed in Bouin’s fluid (Klinipath, Amsterdam, The Netherlands) for 1 h, washed with tap water and stored in 4% formaldehyde solution (Klinipath, Amsterdam, The Netherlands) before embedding in paraffin. The fragment was sectioned into 4 μm thick sections. Every 7th section was analysed for the presence of leukaemic cells after haematoxylin and eosin (HE) staining (Tissue Tek® Prisma™, Sakura, Alphen aan den Rijn, the Netherlands). Per cancer cell line, the entire fragment was sectioned and a total of 105 to 173 sections (representing a tissue surface of 9.3 to 17.4 cm^2^) of GSK1070916 treated tissue were examined by light microscopy for the presence of cancer cell foci by two independent observers.

Isolated tumour cells in suspension were fixated on a slide for immunohistochemical staining using acetic acid and methanol (1:3).

Proliferating cells were identified by immunohistochemical staining with Ki-67 antibody (1:100, DAKO, Agilent Technologies, Santa Clara, CA, USA) while apoptotic cells were highlighted by staining for active caspase-3 (1:200, AC3, BD Biosciences, San Jose, CA, USA). Immunohistochemical staining with polyclonal anti-Aurora B antibody (1:200, Invitrogen, Carlsbad, CA, USA) or polyclonal anti-Aurora C (1:200, Bioss Antibodies, Woburn, MA, USA) was performed to examine AURKB and AURKC expression in ovarian tissue and CML/AML cells. Immunostaining of cytokeratin AE1/AE3 (1:200, DAKO, Agilent Technologies, Santa Clara, CA, USA), a marker for unilaminar follicles [74], was used as a control.

Antigens were retrieved with citrate buffer (ScyTEK Laboratories, Logan, UT, USA). Next, sections were incubated with Vectastain Avidine/Biotine Blocking Kit followed by incubation with normal horse or goat serum (all from Vector Laboratories, Burlingame, CA, USA) depending on the secondary antibody. Subsequently sections were incubated with the primary antibodies followed by incubation with Biotinylated anti-Mouse or anti-Rabbit (both from Vector Laboratories, Burlingame, CA, USA). To visualise the target-antibody interaction Vectastain AB-complex and DAB (both from Vector Laboratories, Burlingame, CA, USA) incubation was used. After counterstaining with haematoxylin (Vector Laboratories, Burlingame, CA, USA), slides were evaluated by light microscopy and photographed using Visiontek® Live Digital Microscope (Sakura, Alphen aan den Rijn, The Netherlands).

### Viability assays of ovarian cortex tissue and follicles

The viability of the ovarian cortex tissue and follicles before and after purging was determined by four different assays: conventional (immuno)histochemistry, a glucose uptake assay [30], Neutral Red staining [47] and in vitro growth (IVG) of small follicles [54, 55]. After thawing, ovarian cortex fragments were treated for 24 h with 1 μM GSK1070916 or solvent and washed. Cortex fragments were immediately used for analyses for glucose uptake assay and IVG assay. For histology and Neutral Red uptake, fragments were incubated for an additional 24 h culture, to allow possible damage to the tissue and follicles to become apparent before analysis. All assays were performed using ovarian cortex tissue from three subjects, except for the IVG assay which was performed in tissue from two subjects.

#### Glucose uptake assay

The glucose uptake assay was performed as described previously [30, 59, 62] and used as a measure of ovarian cortex tissue viability. In brief, ovarian cortex tissue fragments (approximately 1 × 1 × 1 mm) were cultured in DMEM/10% FCS/40 μg/mL Gentamycin. After 4 days, the glucose level in the spent culture medium was measured to determine glucose uptake per hour per mg of ovarian tissue. The glucose uptake assay was performed in four replicates per subject and condition.

#### Morphology of follicles

Follicle damage was assessed by HE staining of 4 μm sections as described previously [33, 40, 59]. Follicles were considered damaged if they had a pyknotic oocyte or granulosa cells, intensely eosinophilic cytoplasm or shrinkage of follicular cells. At least 100 primordial follicles per subject per condition were examined.

#### Neutral Red staining

Viability of follicles was analysed by Neutral Red uptake, a proven and reliable method to quantify follicle survival [4, 46, 47]. In brief, after treatment, ovarian cortex tissue was chopped to small fragments (approximately 0.5 × 0.5 × 0.5 mm) using tweezers and scalpel after which the fragments were incubated in 5 mL Ultraculture (Lonza, Basel, Switzerland) supplemented with Collagenase type IA (1 mg/mL, Sigma-Aldrich, Saint Louis, MO, USA) at 37 °C for 1 h to digest the tissue. The tissue was pelleted and resuspended with McCoy’s 5A medium supplemented with Neutral Red (to a final concentration of 50 mg/mL; Sigma-Aldrich, Saint Louis, MO, USA) for 90 min at 37 °C. A squash preparation was made with the partly dissolved tissue fragments and examined by light microscopy. The ratio between viable (red) and non-viable (colourless) follicles was determined by analysing 100 follicles.

#### In vitro growth assay

An IVG assay was performed to determine capacity of primordial follicles to grow after purging using a modified procedure described as culture step 1 of an in vitro maturation protocol [54]. Excess stromal tissue was removed from ovarian cortex tissue fragments purged with GSK1070916 and control tissue. Next, the tissue was dissected into approximately 1 × 1 × 0.5 mm cubes by using a scalpel and transferred to a 24-well plate containing 2 mL of IVG culture medium per well, consisting of McCoy’s 5a medium, glutamine (3 mM), penicillin G (0.1 mg/mL), streptomycin (0.1 mg/mL), transferrin (2.5 μg/mL), sodium selenite (4 ng/mL), human insulin (10 ng/mL), recombinant human FSH (rhFSH, 1 ng/mL), ascorbic acid (50 μg/mL) (all obtained from Sigma-Aldrich, Saint Louis, MO, USA) and human serum albumin (1 mg/mL, Sanquin Plasma Products, Amsterdam, The Netherlands). The tissue fragments were subsequently cultured for 8 days at 37 °C in humidified air with 5% CO_2_. Half the medium was replaced with fresh medium every other day. After 8 days of culture, the fragments were fixed and processed for (immuno)histochemistry. Non-cultured cortex fragments of the same patient were used as controls. HE-stained sections (4 μm) were evaluated and follicles were categorised according to their stage of maturation as previously described [55, 75].

### Statistical analysis

Graphpad Prism®, version 5.03 for Windows (Graphpad Software Inc., San Diego, CA, USA), was used for statistical analyses. A paired parametric Student’s *t* test was applied and a *P* value <0.05 was considered statistically significant. Data from histology and viability were grouped for statistical analyses.

### Ethical approval

This study was approved by the ethics committee of the Radboud university medical centre, Nijmegen, The Netherlands. Written informed consent was obtained from all patients.

## Results

### Expression of Aurora B and C kinases in CML/AML cells and ovarian cortex tissue

To assess the potential effect of the AURKB/C inhibitor GSK1070916 on myeloid leukaemia metastases in human ovarian tissue, we first used Western blot analysis to examine AURKB and AURKC expression levels in a panel of six myeloid leukaemia cell lines and in human ovarian cortex.

**Fig. 1:**
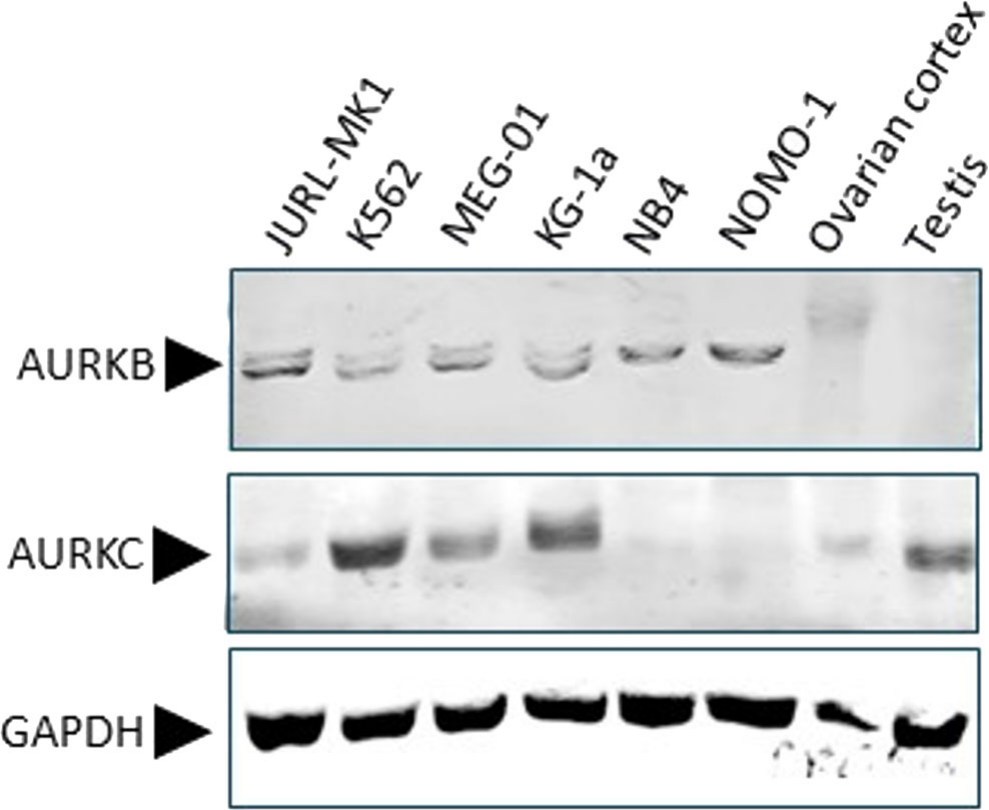
Expression of AURKB and AURKC by Western blot. Expression of AURKB and AURKC examined by Western blot analysis of CML (JURL-MK1, K562 and MEG-01) and AML (KG-1a, NB4 and NOMO-1) cell lines cultured in suspension and human gonadal tissues (ovarian cortex and testicular tissue). GAPDH was used as a loading control. AURKB was detectable in all CML/AML cell lines with as a single protein band of approximately 35 kDa (NB4 and NOMO-1), or in combination with a slightly smaller protein band (JURL-MK1, K562, MEG-01 an KG-1a). AURKB was undetectable in ovarian cortex tissue or testicular tissue. AURKC was detected in JURL-MK1, K562, MEG-01, KG-1a, ovarian cortex tissue and testicular tissue at different expression levels but absent in NB4 and NOMO-1

In suspension culture, all CML/AML cell lines tested expressed similar levels of AURKB. In the AML lines NB4 and NOMO-1, a single immunoreactive protein was detected whereas the other cell lines showed expression of an additional, slightly smaller AURKB protein. AURKC was detected at different expression levels in tumour cell lines JURL-MK1, K562, MEG-01 and KG-1a, but was absent in the AML cell lines NB4 and NOMO-1. AURKB expression was not detected in ovarian cortex tissue or testicular tissue, while AURKC was abundantly expressed in testicular tissue and at low levels in ovarian tissue (Fig. 1).

**Fig. 2:**
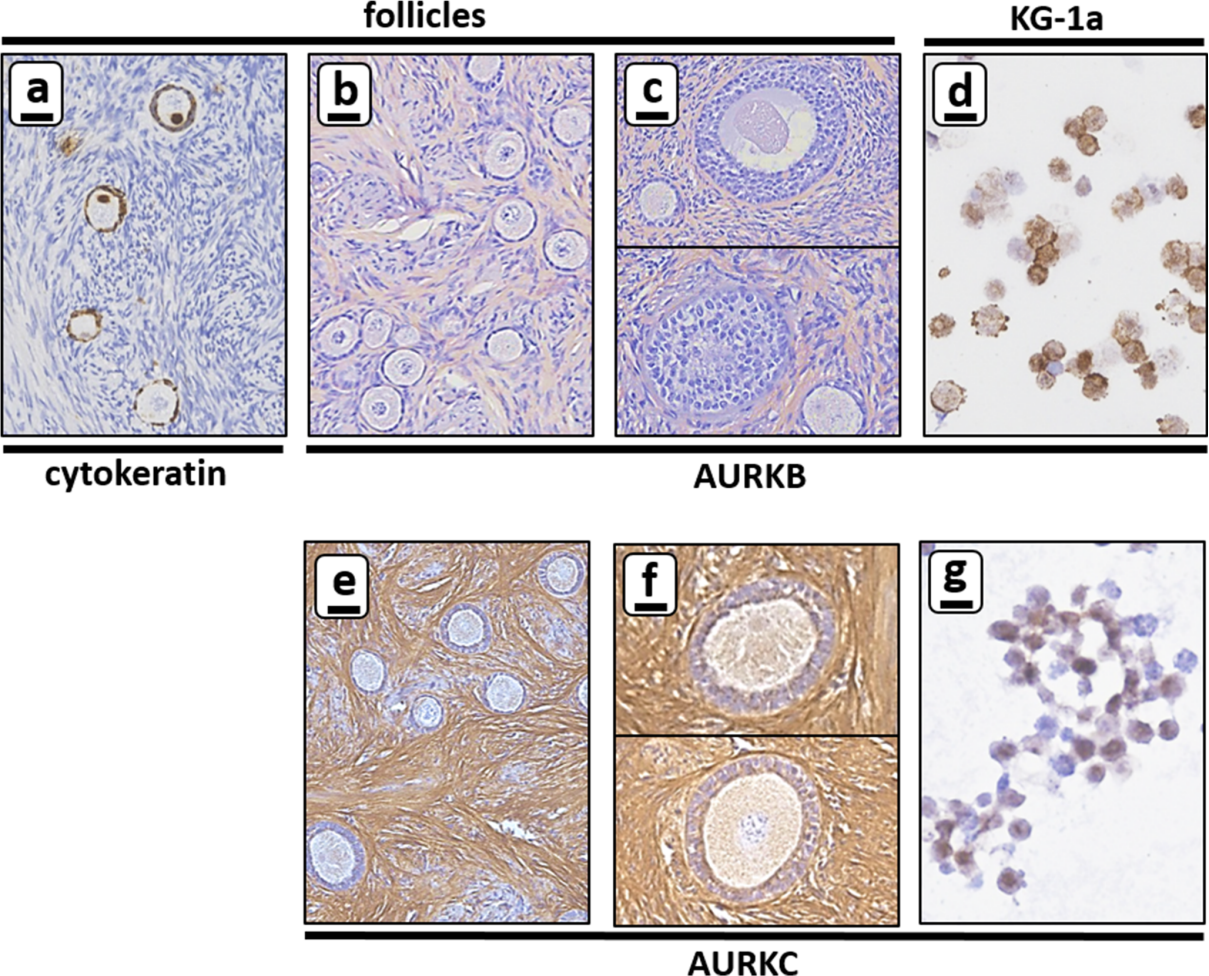
Expression of AURKB and AURKC by immunohistochemistry. Expression of AURKB was examined for all CML and AML cells in suspension and for ovarian cortex tissue by immunohistochemistry with cytokeratin AE1/AE3 as a control for small follicles (panel **a**). AURKB expression was not detectable in the oocytes or granulosa cells of either primordial, primary or secondary follicles (panel **b** and **c**). AURKB expression was detectable in the majority of cells of the CML/AML cell lines in suspension (only shown for the AML cell line KG-1a, panel **d**). Expression of AURKC was examined for CML and AML cells in suspension and ovarian cortex tissue separately. No AURKC expression was detectable in the oocytes or granulosa cell of primordial or primary follicles (panel **e**), but AURKC was present in oocytes of secondary follicles (panel **f**). AURKC expression was clearly detectable in the majority of CML/AML cell lines in suspension (only shown for the AML cell line KG-1a, panel **g**). Scale bars represent 20 μm

Immunohistochemical staining for AURKB and AURKC was performed to confirm the expression profile obtained by Western blot and to further delineate which cell type expressed AURKC in the ovarian cortex tissue (Fig. 2).

The polyclonal antibodies against AURKB and AURKC gave high background signals of extra cellular matrix components between cells, while leaving small follicles unstained (Fig. 2b, c, e and f). AURKB could not be detected in oocytes, granulosa cells of primordial, primary or secondary follicles or ovarian stromal cells. In contrast to AURKB, the oocytes of secondary follicles were positive for AURKC (compare Fig. 2c and f). Expression levels of AURKB and AURKC proteins fluctuate throughout the various stages of mitosis, which is reflected by the difference in immunohistochemical staining intensity of these proteins between individual cells (Fig. 2d and g) [6, 7].

### Purging ovarian cortex tissue by inducing mitotic catastrophe in CML and AML tumour foci

**Fig. 3:**
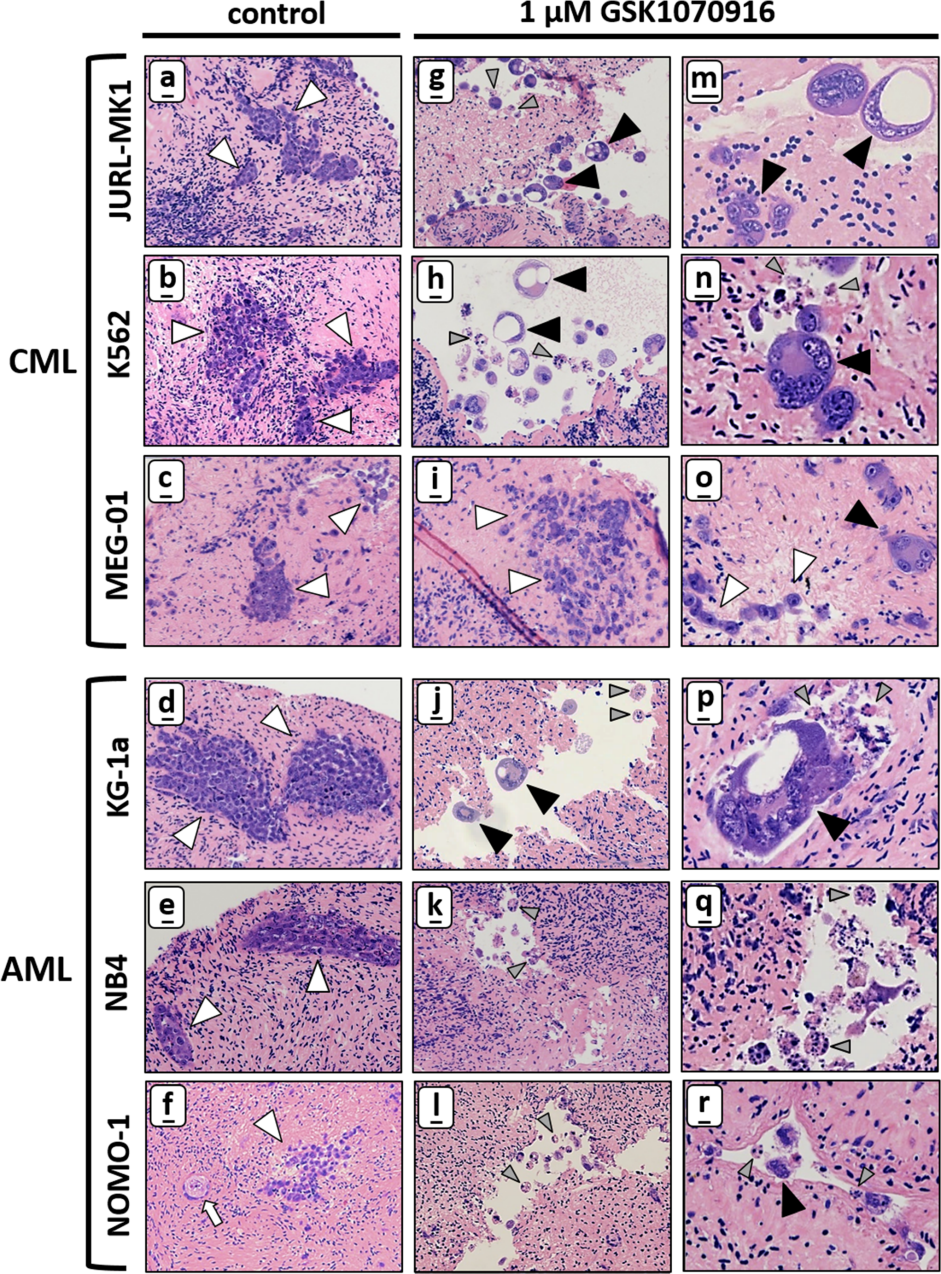
CML and AML tumour foci in ovarian cortex are sensitive to GSK1070916. HE staining showing that CML tumour foci (JURL-MK, K562 and MEG-01) and AML tumour foci (KG-1a, NB4 and NOMO-1) were abundantly present in control human ovarian cortex treated with solvent-only (panel **a**–**f**, white arrowheads indicate tumour foci, the white arrow points at a primordial follicle). After treatment for 24 h with 1 μM GSK1070916 followed by an additional culture for 6 days, tumour foci of JURL-MK1, K562, KG-1a, NB4 and NOMO-1 cells could no longer be detected in ovarian cortex tissue. Large syncytia with sometimes large vacuoles (panel **g**–**r**, black arrowheads indicate syncytia) were present in cortex tissue harbouring JURL-MK1, K562 and KG1-a cells after treatment with 1 μm GSK1070916. Apoptotic bodies (grey arrow heads) were present in all cell lines in ovarian cortex tissue after purging except for MEG-01, whereas NB4 and NOMO-1 showed almost exclusively apoptotic bodies with few small syncytia (panel **q**–**r**). In contrast to the other five cell lines, MEG-01 showed mostly tumour foci harbouring morphologically normal tumour cells (white arrowheads in panel **i** and **o**) next to few small syncytia with up to four nuclei after treatment (black arrowhead in panel **o**). Scale bars represent 20 μm in panel **a**–l and 10 μm in panel **m**-**r**

Tissue sections derived from cultured human ovarian cortex fragments micro-injected with either CML or AML cells all showed efficient formation of numerous small tumour masses throughout the cortex fragment (Fig. 3a–f). Identification of CML/AML cells in ovarian cortex tissue was relatively straightforward based on the large size of the leukaemic cells, shape and HE-staining intensity. The size of tumour foci and the degree of cell dissemination varied considerably between cell lines. JURL-MK1, K562 and KG-1a all formed both small and large tumour foci of tightly packed cells whereas MEG-01, NB4 and NOMO-1 only formed small foci. K562, MEG-01, NB4 and NOMO-1 tumours showed extensive dissemination in the surrounding tissue (Table 2).

**Table 2:** Characteristics of CML/AML tumour cells in ovarian cortex tissue. Growth characteristics of CML (JURL-MK1, K562 and MEG-01) and AML (KG-1a, NB4 and NOMO-1) tumour cells in ovarian cortex tissue 6 days after a 24 h treatment with 1 μM GSK1070916 or solvent-only (control)

cell line	control	1 μM GSK1070916
JURL-MK1	small and large foci of tightly packed cells, some dissemination	many small syncytia with up to 11 nuclei, apoptotic bodies
K562	small and large foci of tightly packed cells, extensive dissemination	many large syncytia with up to 30 nuclei, apoptotic bodies
MEG-01	small non-compact foci, extensive dissemination	mainly single cells and very few small syncytia with 2-4 nuclei
KG-1a	small and large foci of tightly packed cells, some dissemination	many large syncytia with up to 30 nuclei, apoptotic bodies
NB4	small foci, extensive dissemination	mainly apoptotic bodies and very few small syncytia with up to 6 nuclei
NOMO-1	small foci, extensive dissemination	mainly apoptotic bodies and very few small syncytia with up to 7 nuclei

We investigated the purging capacity of a 24 h exposure to the AURKB/C inhibitor GSK1070916 of ovarian cortex tissue fragments harbouring CML or AML tumour foci. In the control tissue treated for 24 h with solvent-only all tumour cell lines displayed abundant numbers of tumour foci throughout the tissue fragment (Fig. 3a–f). The tumoricidal effect of GSK1070916 was determined by exposing ovarian cortex fragments with CML or AML tumour foci to 1 μM GSK1070916 for 24 h, followed by extensive washing to remove the inhibitor. The treated fragments were cultured for an additional 6 days before analysis to allow any remaining cancer cells to form new foci. To confirm the absence of morphologically normal tumour cells, serial sections of GSK1070916 treated tissue of all cell lines all in three different subjects was examined histologically. For each cell line, a total of 105 to 173 tissue sections, representing 9.3 to 17.4 cm^2^ of ovarian cortex tissue surface, was examined.

All cell lines reacted differently to GSK1070916 exposure. Whereas JURL-MK1 showed many syncytia with a diameter up to 40 μm containing up to 11 nuclei (Fig. 3g and m), K562 and KG-1a displayed even larger syncytia (≤ 80 μm) with up to 30 nuclei (Fig. 3h, j, n and p). Tumour foci of AML cell lines NB4 and NOMO-1 were almost exclusively reduced to apoptotic bodies with only few small syncytia (Fig. 3k, l, q and r). The megakaryoblast leukaemia cell line MEG-01 derived tumour foci appeared to be less sensitive to GSK1070916 treatment since only very few small syncytia could be detected among large numbers of morphologically normal MEG-01 cells (Fig. 3i and o).

**Fig. 4:**
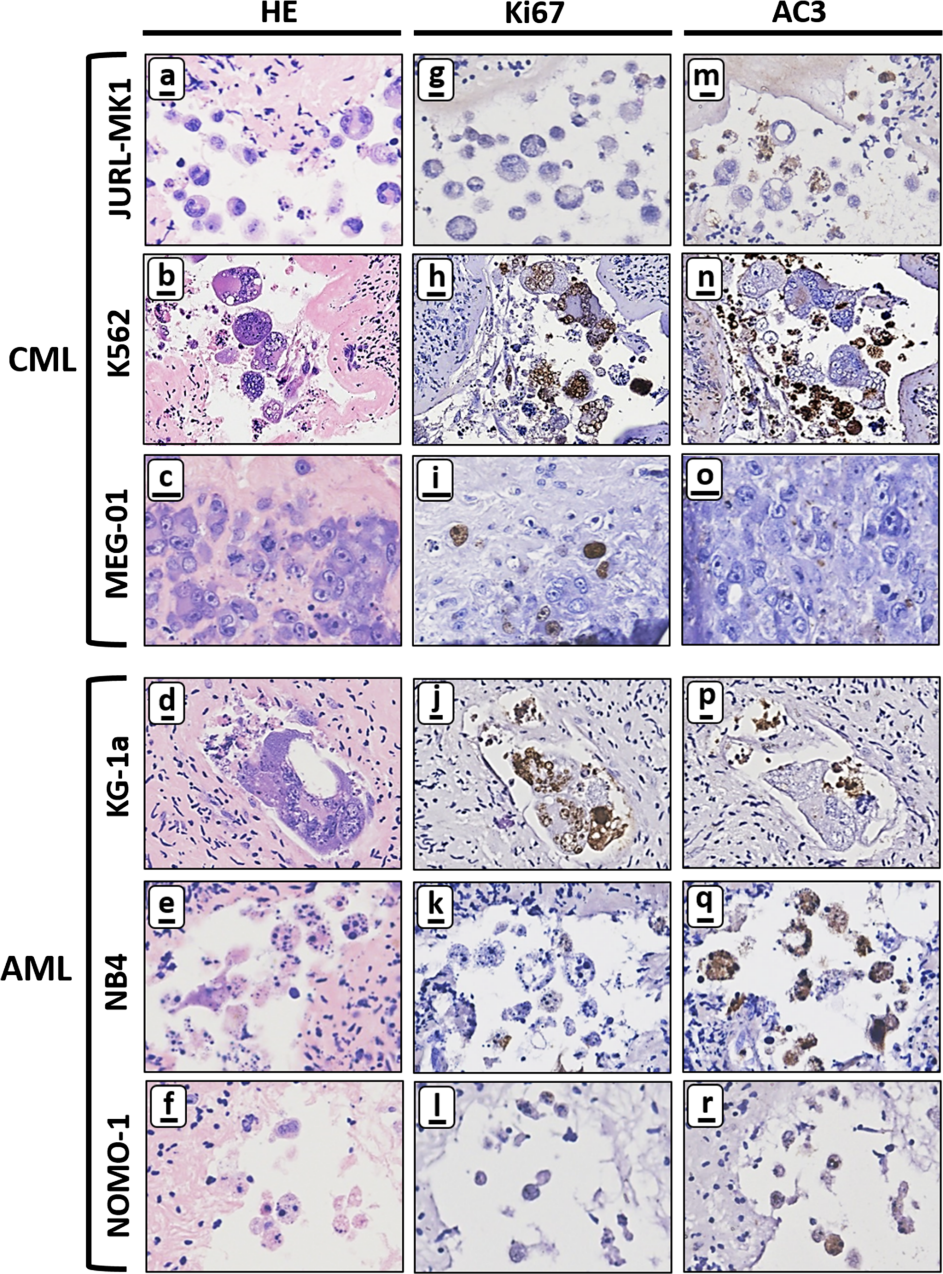
Immunohistochemical analysis of ovarian cortex tissue harbouring CML and AML tumour foci after purging for 24 h with 1 μM GSK1070916. Large-scale formation of syncytia and apoptotic bodies in human ovarian tissue containing CML (JURL-MK1 and K562) and AML (KG-1a, NB4 and NOMO-1) tumour foci after 24-h treatment with 1 μM GSK1070916 followed by 6 days of culture. Nuclei of intact syncytia were positive for proliferation marker Ki-67 (panel **g**–**j**) whereas the degraded syncytia and surrounding apoptotic bodies were positive for the apoptotic marker anti-active caspase 3 (AC3) (panel **m**–**r**). Note that MEG-01 cells did not show extensive formation of syncytia or apoptotic bodies (panel **c**) with cells being positive for Ki-67 (panel **i**) but negative for AC3 (panel **o**). Scale bars represent 10 μm except in panel **b**, **h** and **n** where it represents 25 μm

Next, we investigated the presence of viable and apoptotic tumour cells in GSK1070916 treated tissue by immunohistochemical staining for the nuclear proliferation marker Ki-67 and the apoptosis marker AC3. Most syncytia were positive for Ki-67, suggesting that in these aberrant multinuclear structures part of the processes associated with cell proliferation are still active (Fig. 4g, h, j–l). Interestingly, syncytia harbouring Ki-67-positive nuclei do not show apoptosis as indicated by the absence of AC3 staining (compare Fig. 4h, n, j and p). Most of the smaller structures directly adjacent to the intact syncytia were positive for AC3, indicating large scale apoptosis.

The MEG-01 tumour foci reacted very differently to GSK1070916 treatment compared to the other cell lines as judged by the presence of histologically normal and mitotically active cells without any evidence for apoptosis (Fig. 4c, i and o).

### Purging ovarian cortex tissue by inducing mitotic catastrophe in primary AML cells

**Fig. 5:**
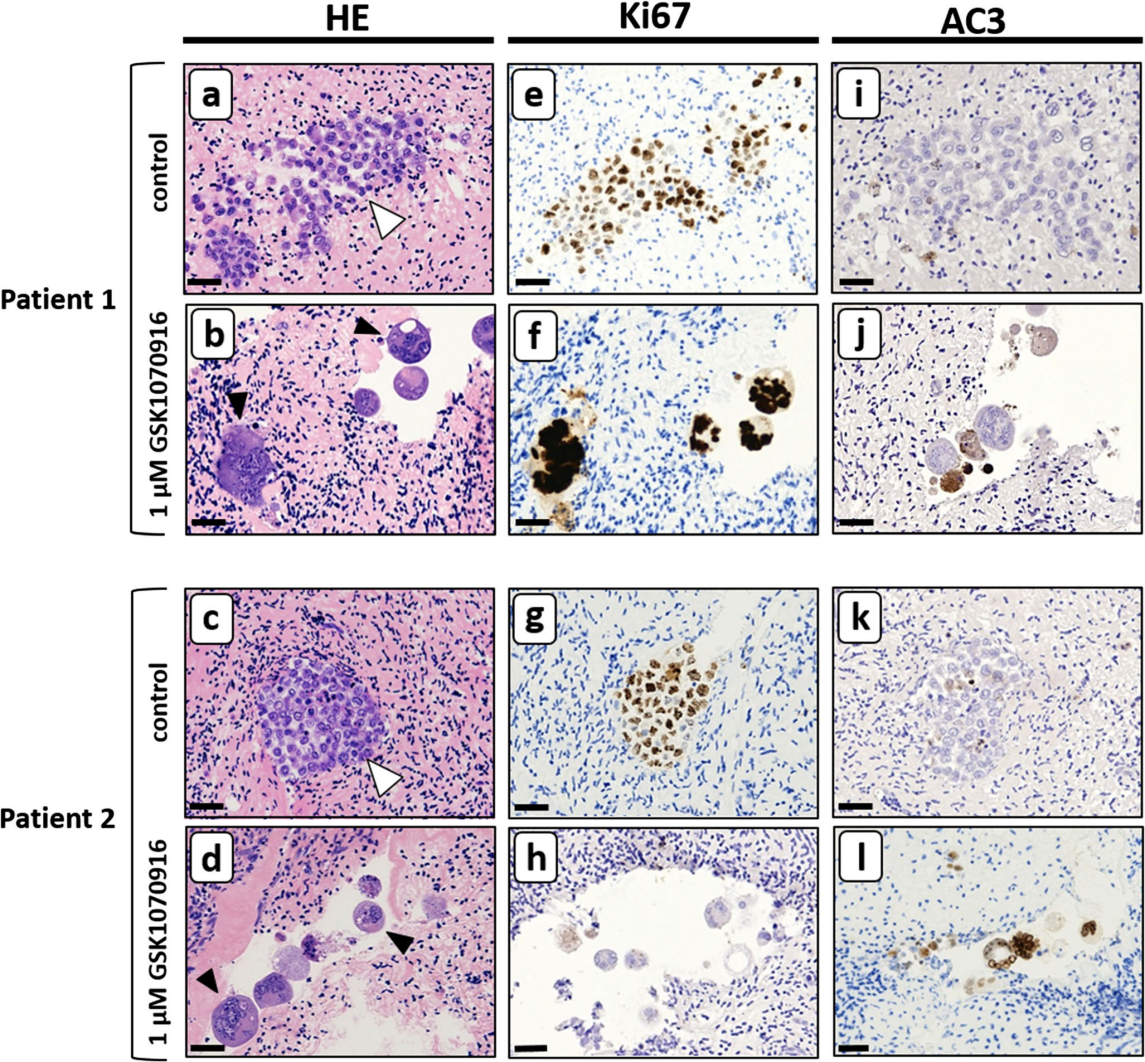
Primary AML tumour foci in ovarian cortex are sensitive to GSK1070916. Experimentally induced tumour foci of primary AML cells in human ovarian cortex tissue treated with solvent only showed normal cell morphology (panel **a** and **c**; white arrowheads indicate tumour foci) with a substantial part of the AML cells positive for the proliferation marker Ki67 (panel **e** and **g**) and very few cells positive for the apoptotic marker active caspase-3 (AC3, panel **i** and **k**). After treatment for 24 h with 1 μM GSK1070916 foci of normal tumour cells could no longer be detected and AML cells gave rise to large syncytia containing multiple nuclei and sometimes large vacuoles (panel **b** and **d**, black arrowheads indicate syncytia). Most nuclei of intact syncytia were positive for the proliferation marker Ki-67 (panel **f** and **h**) whereas degrading syncytia and apoptotic bodies were positive for the apoptotic marker active caspase-3 (panel **j** and **l**). Scale bars represent 30 μm. In supplemental Fig. 1, the results of purging primary cells from ovarian cortex tissue of two additional AML patients are shown

To confirm the tumoricidal effect on primary AML cells, the purging experiment was repeated with primary cells of four AML patients. Similar to the cell lines, the primary AML cells formed both small and large tumour foci of tightly packed cells with dissemination in the surrounding tissue when treated for 24 h with solvent-only (Fig. 5a and c). In these tumour foci, a large number of cells were positive for the proliferation marker Ki67 (Fig. 5e and g), while only few apoptotic cells were observed (Fig. 5i and k). After the exposure to 1 μM GSK1070916 for 24 h, the primary AML cell foci of all four patients showed formation of both large and small syncytia (up to 28 nuclei, Fig. 5b and d) with signs of extensive apoptosis in some syncytia (Fig. 5j and l), while other syncytia contained numerous nuclei that were positive for Ki67 (Fig. 5f, note that the histology in two patients is shown. See supplementary Fig. 1 for the histology in the other two patients). To confirm the absence of morphologically normal tumour cells, serial sections of GSK1070916 treated tissue were examined histologically and no foci of morphologically normal tumour cells could be detected.

### Inhibition of AURKB/C has no effect on ovarian tissue viability or follicular integrity

The effect of the 24 h purging protocol with 1 μM GSK1070916 on viability and follicular integrity of ovarian cortex tissue was examined using four different assays.

#### Glucose uptake assay

Metabolic activity of ovarian cortex tissue from two out of three subjects was not affected by treatment with GSK1070916 as glucose uptake was statistically not different from tissue exposed to solvent only. In the tissue from the third subject, a slight but statistically significant difference in glucose uptake in favour of the GSK1070916 treated tissue was observed (Table 3). In line with previous reports [30, 59, 68], glucose uptake by ovarian cortex tissue in vitro is variable between subjects ranging from 5.675 to 9.050 nmol/mg tissue/h.

**Table 3:** Results glucose uptake assay

Glucose uptake (nmol/mg tissue/h)			
	Mean		
	1 μM GSK1070916	Control	(95% CI)
Subject 1	9. 050	6.100	−2.410 to −0.1397
Subject 2	6.275	7.550	−2.052 to 1.102
Subject 3	5.675	6.150	−0.5102 to 6.410

In vitro glucose uptake by cortical ovarian tissue after 24-h treatment with 1 μM GSK1070916 or control solvent-only determined in quadruplicate per subject and condition. No significant difference is observed between the control and the fragments treated with the inhibitor in two out of three subjects. In subject 1, there is a slight but statistically significant difference in favour of the tissue treated with 1 μM GSK1070916.*CI, confidence interval

#### Morphology of follicles

**Fig. 6:**
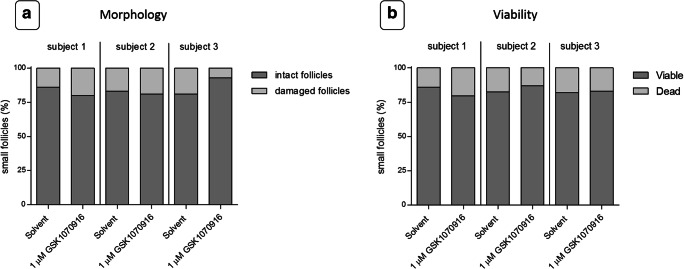
Morphology and viability of follicles. Ovarian cortex tissue was exposed to a 24-h ex vivo treatment of solvent-only or 1 μM GSK1070916, washed and cultured for an additional 24 h, to allow tissue damage to become apparent. At least 100 follicles were evaluated per patient per condition. **a** Graphical depiction of the percentage of intact and damaged follicles after treatment. Morphology was examined by HE staining. No statistically significant difference (*P* = 0.83) was observed between follicles from the control or the tissue treated with the inhibitor. **b** Graphical depiction of the percentage of viable Neutral red positive follicles and non-viable Neutral red negative follicles. Ovarian cortex tissue was examined by Neutral red staining. No statistically significant difference (*P* = 0.92) was observed between follicles from the control or the tissue treated with the inhibitor

Morphological examination of ovarian follicles with standard HE stained sections from ovarian cortex treated with 1 μM GSK1070916 showed no statistically significant difference regarding the percentage of normal follicles compared with the control (*P* > 0.05, Fig. 6a). The majority of follicles were in the primordial stage.

#### Neutral red uptake

In addition to follicular morphology, we examined the effect of GSK1070916 on the viability of preantral follicles in ovarian cortex tissue by Neutral red staining (Fig. 6b). The percentage of viable Neutral red positive follicles was not different between the fragments treated with 1 μM GSK1070916 or solvent (*P* > 0.05).

#### In vitro growth

**Fig. 7:**
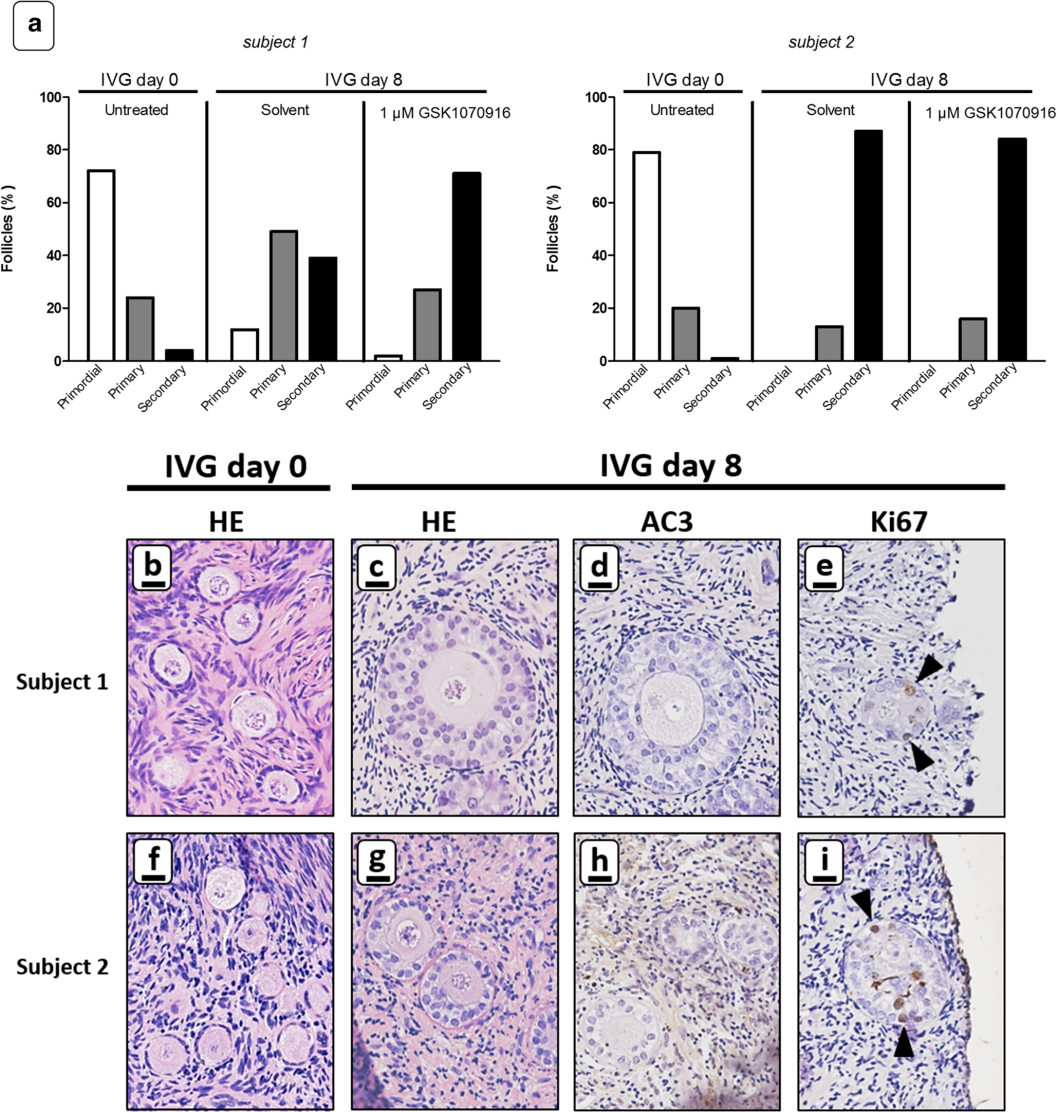
In vitro growth of follicles after GSK1090716 exposure. Ovarian cortex tissue of two subjects was exposed to 1 μM GSK1070916 or solvent-only for 24 h, washed and subsequently cultured in IVG culture medium for 8 days. **a** Graphical depiction of percentage of primordial, primary and secondary follicles in ovarian cortex tissue of two subjects in uncultured tissue (control) and tissue subjected to IVG for 8 days after a 24 h exposure to 1 μM GSK1070916 or solvent only. At least 100 follicles were evaluated per sample. Standard HE staining showed mainly primordial follicles in uncultured ovarian cortex tissue (panel **b** and **f**) and a substantial increase in secondary follicles after IVG for 8 days irrespective of previous GSK1070916 treatment (panel **c** and **g**). Immunohistochemistry with AC3 indicated apoptosis was absent in oocytes or granulosa cells in GSK1070916 treated tissue (panel **d** and **h**) whereas Ki-67 positive granulosa cells showed ongoing folliculogenesis in secondary follicles (panel **e** and **i**, black arrowheads point to Ki-67 positive granulosa cells). Scale bars represent 20 μm

Ovarian cortex tissue of two subjects was exposed to a 24h ex vivo treatment of 1 μM GSK1070916 or solvent-only and subsequently cultured for 8 days with IVG culture medium. Follicles were evaluated by standard HE staining and by immunohistochemistry for AC3 and Ki-67. Compared to the uncultured fragments (Fig. 7a and e), follicular growth could be clearly observed in fragments treated with solvent and in the GSK1070916 treated fragments, as illustrated by a shift from primordial to primary and secondary follicles after 8 days of IVG (Fig. 7b, f and i). GSK1070916-treated fragments showed no signs of apoptosis as illustrated by lack of AC3 expression in oocytes and granulosa cells after culture (Fig. 7c and g). Ki-67-positive granulosa cells could be detected in follicles of GSK1070916 treated tissue fragments indicating granulosa cell proliferation was still ongoing at day 8 of IVG (Fig. 7d and h).

## Discussion

Autotransplantation of cryopreserved ovarian cortex tissue remains a procedure that harbours the risk of reintroducing the cancer to the cured patient through malignant cells that may be present in the graft. Standard screening methods of ovarian cortex tissue prior to autotransplantation involve immuno-histological analysis and tumour-specific PCR examination, sometimes combined with xenotransplantation to immunodeficient mice, multicolor flow cytometry or next generation sequencing [15, 64, 72, 83]. Since the analysed tissue fragment is no longer available for autotransplantation, these screening methods do not assure that the remaining fragments are devoid of malignant cells. Tissue fragments of the same ovary were shown to give different results when tested for the presence of malignant cells by tumour-specific PCR leading to sampling bias [5, 9, 65]. To circumvent this problem, several strategies aimed at separating the ovarian follicles from the potentially contaminated stromal compartment before transplantation are currently being pursued [2, 48, 54, 63, 78]. However, these methods are not yet clinically available and rely on dissociation of the tissue by separating the follicles from their naturally surrounding stromal cells. Folliculogenesis depends heavily on the interaction of follicles with the surrounding tissue and dissociation may have an impact on follicle maturation after transplantation. To avoid problems caused by sampling bias or tissue dissociation, we recently developed a protocol based on pharmacological treatment to purge human ovarian cortex tissue of malignant cells without compromising ovarian tissue or follicular integrity. Rhabdomyosarcoma tumour foci were completely eliminated from ovarian tissue by a 24h ex vivo treatment using Verteporfin, an inhibitor of YAP/TAZ oncoproteins [36, 49, 59, 77]. Purging of ovarian cortex tissue therefore offers a potentially safe strategy to eliminate malignant cells from all tissue fragments prior to autotransplantation without affecting tissue viability.

In the current study, we have extended our efforts by developing efficient purging protocols for other clinically relevant malignancies. We investigated the use of GSK1070916, an onco-suppressive agent that induces mitotic catastrophe by driving cells to an irreversible antiproliferative fate of death [35, 52, 58, 80]. GSK1070916 exerts this effect by inhibiting AURKB/C and inhibits malignant cell proliferation in a wide range of tumour cell lines in vitro and in xenograft models of human tumours [35]. Aurora kinase inhibitors have not been approved for systemic cancer treatment in humans and only a Phase I clinical trial has been conducted with GSK1070916 [7, 56]. Neutropenia caused by Aurora B toxicity was found to be the limiting factor in achieving an effective systemic treatment in a clinical setting [44, 45, 56]. Obviously, this is of no concern to the current study in which ovarian cortex tissue is purged from malignant cells ex vivo, in the absence of perfusion.

CML and AML cell lines were selected to represent malignancies that are clinically relevant and an indication for OTC [14]. AML is one of the most common forms of haematological malignancies that occurs in girls and young women and requires immediate start of anti-cancer treatment with fertility threatening alkylating agents, sometimes followed by haematopoietic stem cell transplantation which further increases the risk of infertility [67]. Ovarian tissue fragments from patients with CML and AML have a high chance of being contaminated with malignant cells and ovarian cortex from a CML patient has actually been shown to transfer the disease upon xenotransplantation to immunodeficient mice [1, 3, 17]. Although ovarian cortex fragments of leukaemia patients are the most suitable for our studies, they are not available in the quantity required for purging experiments. Furthermore, because of sampling bias, it is impossible to predict which cortex fragments will actually contain metastases without a prior analysis that will render the fragment unsuitable for ex vivo purging. Moreover, cortex fragments of the same ovary may differ with respect to the presence of malignant cells, with some fragments being positive while the others were not [9, 65]. Cryopreserved ovarian cortex fragments from cancer patients might therefore be less suitable for purging experiments because it is not possible to determine the efficiency of purging when we are unable to establish the presence of malignant cells in the tissue before treatment.

To analyse the effect of GSK1070916 on metastasised CML and AML cells, we therefore used an established tumour model based on the micro-injection of cancer cells (both AML and CML cell lines and primary AML cells) into human ovarian cortex tissue followed by several days of culture to allow malignant cells to form small metastases [59, 61]. We first opted for cell lines since the cancer-type specific mutations are present in these cells and their long-term proliferation potential in vitro was required for our experiments. Furthermore, cell lines are widely and successfully used in cancer research and anti-cancer drug discovery [51]. To confirm our findings with the cell lines, we expanded our experiments using primary cancer cells which may represent the in vivo situation more closely.

The sensitivity of cancer cells to pharmacological inhibition depends on the rigidity and specific extra-cellular components of their microenvironment [25, 50, 59]. Our approach allowed us to test the inhibitory effect of GSK1070916 on leukaemic cells in the appropriate microenvironment. The ex vivo treatment was limited to 24 h in order to minimise culture induced activation of primordial follicles and subsequent follicular burn-out after autotransplantation [28, 55]. In vitro activation of small follicles has been shown to require special culturing conditions including ovarian fragmentation and Akt pathway stimulation [37, 38]. In addition, a 48-h culture period of ovarian cortex tissue prior to transplantation has resulted in live birth [37], suggesting that the short ex vivo culture used in the current study is unlikely to lead to extensive activation of follicles. Moreover, follicles in control tissue cultured for up to 10 days did not show any obvious signs of activation.

After purging, the entire cortex fragment was sectioned and analysed for the presence of morphologically normal tumour cells. Our extensive histological analysis revealed that tumour foci of both CML (JURL-MK1, K562) cells, AML (KG-1a, NB4 and NOMO-1) cells and primary AML cells were completely eliminated by a 24h treatment with 1 μM of GSK1070916, leaving only large multinuclear syncytia and apoptotic bodies. Inhibition of Aurora B is known to cause defective mitosis and cytokinesis failure leading to large polyploid syncytia and ultimately to apoptosis [35, 52, 58, 80].

This is in line with our immunohistochemical staining results for the proliferation marker Ki-67 and apoptosis marker AC3. Syncytia from both the cell lines and the primary cells were negative for AC3 but still expressed Ki-67, indicating that these cells were not arrested in mitosis but failed to divide. The Ki-67 negative/AC-3 positive small cellular bodies next to the syncytia with eosinophilic cytoplasm and condensed chromatin are most probably syncytia in the process of apoptosis. Strikingly, the number of nuclei (up to 30 nuclei per syncytia in the cell lines and 28 nuclei in the primary cells) observed in histological sections of syncytia was frequently higher than expected considering a 6-day culture period after purging and the 30–50 h doubling time of the CML and AML cells in suspension culture. This suggests that mitosis, in the absence of cytokinesis, of these cells is accelerated after treatment with GSK1070916, leading to the formation of more nuclei than under normal growth conditions.

The acute megakaryocytic leukaemia cell line MEG-01 was largely unresponsive to purging with GSK1070916. Megakaryocytes undergo polyploidization prior to the formation of a large number of platelets and subsequent release into the bloodstream. Downregulation of Aurora B during late anaphase of the endomitotic cycle has been implicated in polyploid formation [39, 81] and is therefore most likely the reason that the MEG-01 cells did not show extensive formation of syncytia or apoptosis after GSK1070916 treatment [32, 76]. Furthermore, cell lines with a polyploid phenotype and high chromosome number are associated with resistance to GSK1070916 [58]. Stratification by phenotyping and karyotyping malignant cells of patients could therefore serve as resistance-associated marker for prediction of response to purging with GSK1070916.

Western blot analysis of AURKB/C expression in CML/AML cell lines revealed AURKC and two AURKB proteins, with a slight difference in molecular weight, for JURL-MK1, K562, MEG-01 and KG-1a. The AML cell lines NB4 and NOMO-1 only expressed the larger AURKB protein but no AURKC. Aurora kinases are subjected to alternative splicing and different functions have been ascribed to the splice variants [26, 79]. Possibly, differences in the AURKB/C protein expression profile are related to their reaction to purging with GSK1070916 as NB4 and NOMO-1 showed mostly apoptosis and did not show the large scale formation of syncytia as observed in JURL-MK1, K562 and KG-1a cell lines.

In this study, we examined purging for 24 h with 1 μM GSK1070916 on CML/AML metastases of cell lines and primary cells in ovarian cortex by extensive histological examination after serial sectioning the entire treated tissue fragments to ensure no normal tumour cells were present. This clearly exceeds the standard clinical pathological examination involving only a limited number of sections of ovarian cortex prior to autotransplantation. Ideally, we would have confirmed the absence of CML/AML tumour cells by sensitive molecular techniques. We have shown previously effective ex vivo purging of ovarian cortex tissue from contaminating alveolar rhabdomyosarcoma with Verteporfin could be confirmed by an RT-PCR for the presence of the PAX3-FOXO1 transcript [59]. However, in contrast to Verteporfin, purging with GSK1070916 not only leads to large scale apoptosis but initially also leads to the formation of syncytia. Although these large multinuclear cells will ultimately go into apoptosis [35, 52, 58, 80], they are metabolically still active at the end of the culture period and therefore probably contain detectable tumour-specific transcripts. Long-term culture of the purged ovarian cortex fragments beyond day 6 to allow any remaining syncytia to be eliminated through apoptosis has been shown to result in significant loss of viability of ovarian tissue and is therefore not reliable [61]. Xenografting to immunodeficient mice could further substantiate the absence of viable tumour cells in purged ovarian cortex tissue.

In addition to its oncosuppresive properties, GSK1070916 should obviously have no compromising effects on the ovarian cortex tissue to maximise the chances of a successful pregnancy after autotransplantation. Our purging protocol with GSK1070916 did not affect overall tissue viability, follicle morphology, follicle viability or the capacity of small unilaminar follicles to develop into secondary follicles. This was not unexpected, as isolated ovarian cortex tissue is essentially mitotically and meiotically silent and not dependent on AURKB/C function.

To determine the capacity of primordial follicles to grow after purging, we used the first culture step of a recently published multi-step in vitro maturation protocol [54]. Culturing the tissue beyond the first step was not performed since growth of small follicles to secondary/multi-laminar follicles is already observed after this first step while the remaining steps of the procedure are technically challenging and lead to morphologically abnormal cumulus oocyte complexes [54].

Expression of Aurora B was not detected in ovarian cortex tissue, either by Western blot or immunohistochemistry. Aurora C was absent from small follicles and only detectable in oocytes at more advanced stages of follicular development. Inhibition of Aurora B/C is therefore unlikely to influence the capacity of ovarian cortex tissue to restore fertility, as this is thought to depend mainly on the pool of small follicles [22, 41]. Restoration of ovarian activity and subsequent recruitment of these follicles after autotransplantation takes between 3.5 and 6.5 months [21]. An effect on these newly recruited follicles by any remaining GSK1070916 in the graft is not to be expected in view of its low molecular weight (0.5 kDa), promoting rapid removal during the extensive washing steps following the purging procedure and massive dilution of the inhibitor directly after transplantation in the surrounding patients’ fluids and tissues.

In mice, Aurora C is essential for proper meiotic cell division during spermatogenesis. Female *Aurkb* and *Aurkc* single or double knockout female mice, however, are still fertile [60]. In humans, homozygous *AURKC* mutation is associated with male infertility, but women carrying this mutation were reported to be fertile [11]. This indicates that, in contrast to spermatogenesis, oogenesis might not require AURKC. Taken together this indicates our ex vivo purging protocol is unlikely to impair the function of the ovarian cortex tissue after autotransplantation.

The results in this paper indicate that purging by targeting the mitotic catastrophe signalling pathway in CML and AML metastases-contaminated ovarian cortex tissue intended for fertility preservation purposes is possible by a short-term incubation with GSK1070916, a specific AURKB/C inhibitor. In addition to the techniques already available to prevent reseeding of CML/AML through transplantation of contaminated ovarian tissue, purging with GSK1070916 may further contribute to the safety of fertility restoration in leukaemia patients currently considered to be at high risk for ovarian involvement.

## Supplementary information

ESM 1 (PNG 4032 kb).

High Resolution Image (TIF 2274 kb).

## Data Availability

Not applicable.
