# Supplementary figures and images for: Purging human ovarian cortex of contaminating leukaemic cells by targeting the mitotic catastrophe signalling pathway
Source: J Assist Reprod Genet. 2021 Mar 16;38(6):1571–88. doi: 10.1007/s10815-021-02081-9 (PMC8266964; doi:10.1007/s10815-021-02081-9)

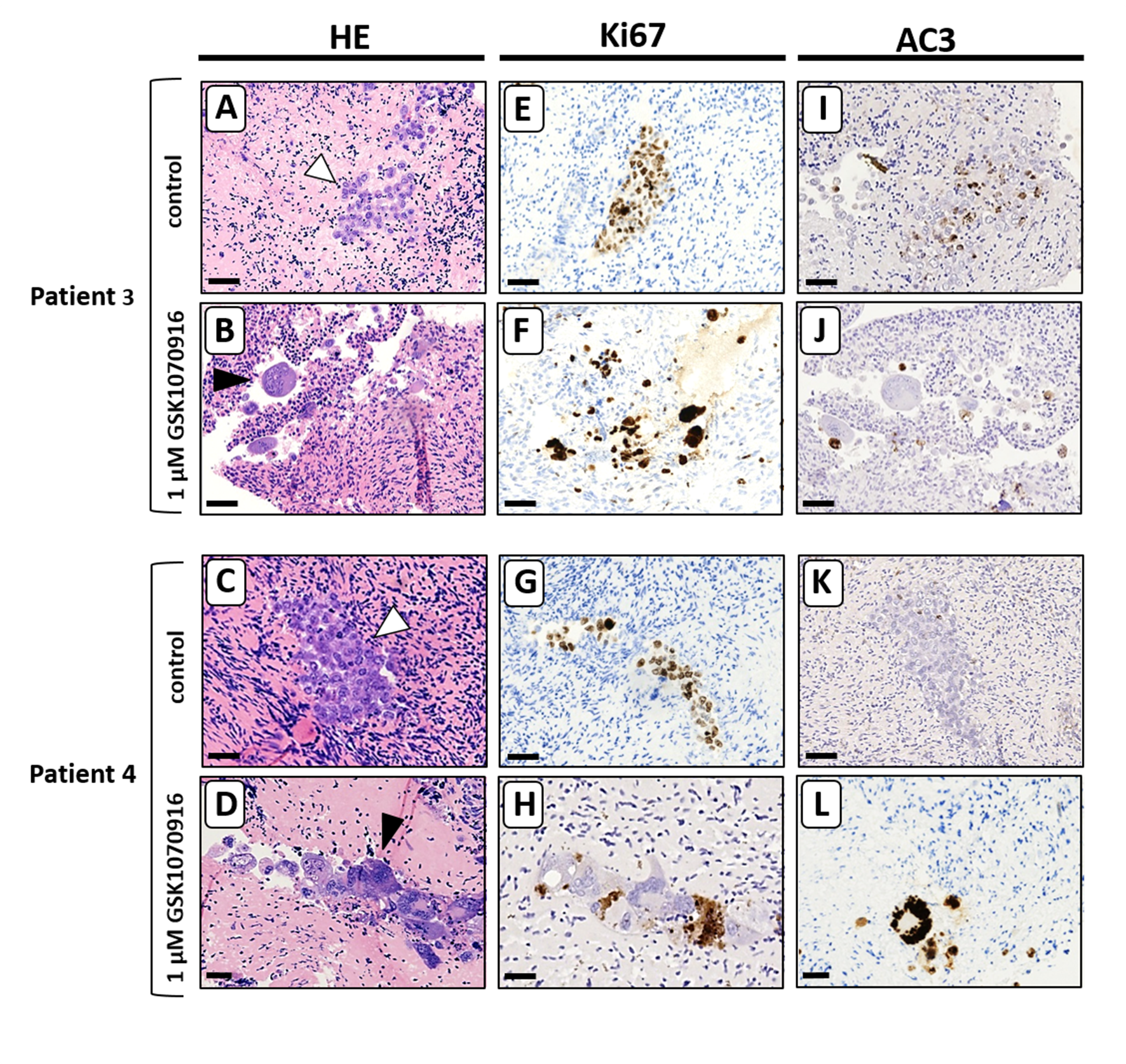

Supplement: Supplementary file 1 — (PNG 4032 kb). [file 10815_2021_2081_Fig8_ESM.png]

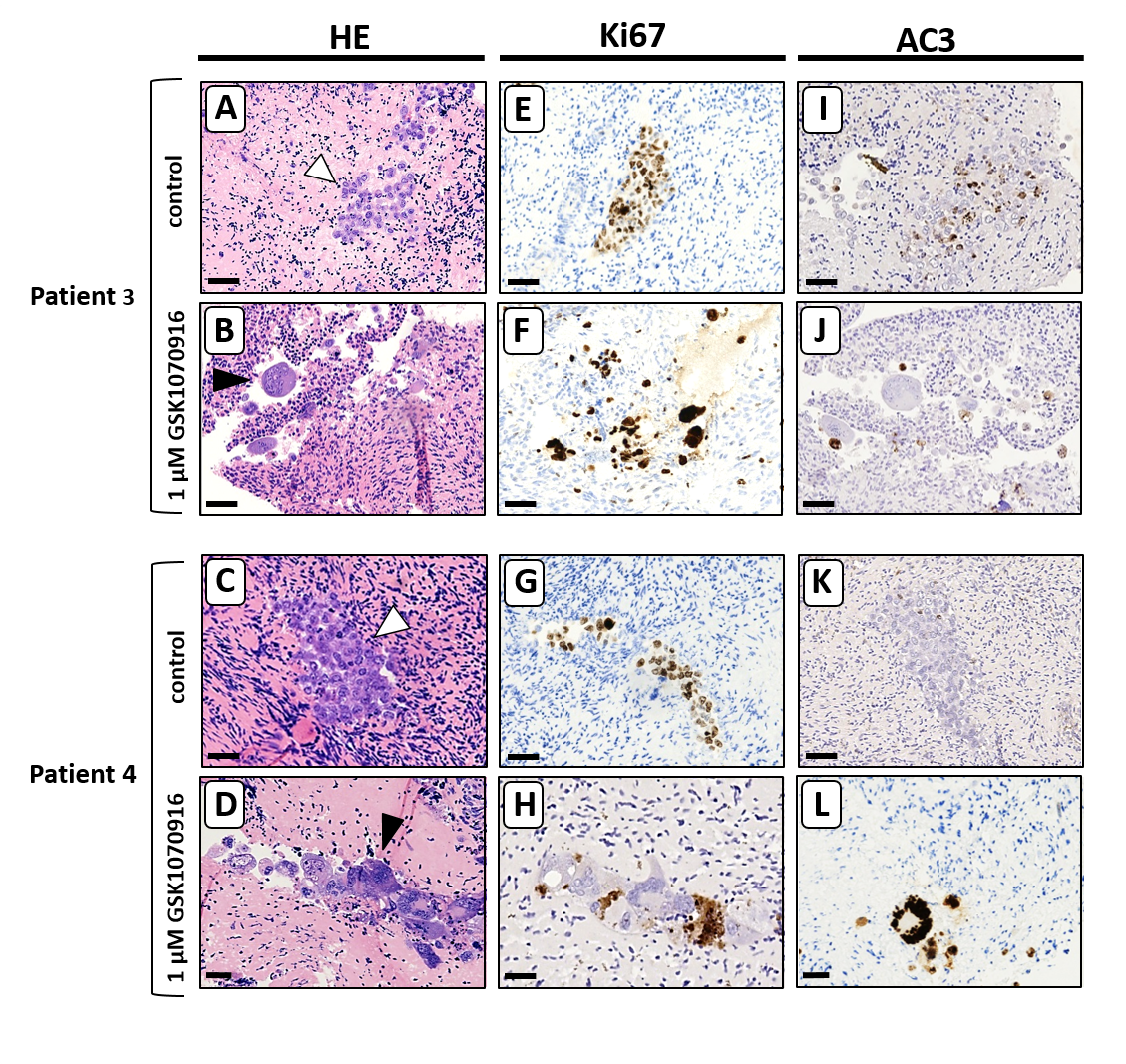

Supplement: Supplementary file 2 — High Resolution Image (TIF 2274 kb). [file 10815_2021_2081_MOESM1_ESM.tif]
